# Superficial Temporal Artery True Fusiform Aneurysm With Several Lateral Feeding Vessels

**DOI:** 10.7759/cureus.13973

**Published:** 2021-03-18

**Authors:** Dieter Brummund, Angela Chang, Obteene Azimi-Ghomi, Brandon Diaz, Harry Sendzischew

**Affiliations:** 1 Department of General Surgery, Aventura Hospital and Medical Center, Aventura, USA; 2 Department of Anesthesiology, Aventura Hospital and Medical Center, Aventura, USA; 3 Department of General Surgery, Mount Sinai Medical Center, Miami Beach, USA

**Keywords:** superficial temporal artery, fusiform aneurysm, true aneurysm, flight attendant, superficial temporal artery aneurysm, aneurysm branching

## Abstract

A 49-year-old man flight attendant with a past medical history of Roux-en-Y bypass and massive weight loss 18 months prior was referred for surgical management of a superficial temporal artery aneurysm. Imaging confirmed the diagnosis. Intraoperatively a 1 cm fusiform aneurysm was identified with numerous feeding side branch vessels. The aneurysm was suture ligated and excised in toto with pathologic analysis showing involvement of all vessel layers. This case highlights a rare true aneurysm of the superficial temporal artery and aberrant anatomy of multiple side branches feeding the aneurysm and complicating dissection and excision.

## Introduction

Aneurysms are defined as pathologic expansion of a vessel's diameter greater than 1.5 times. They may be fusiform involving the body of the artery, or saccular occurring at a branch point. True aneurysms involve all layers of the vessel wall as compared to pseudoaneurysms, in which the aneurysmal expansion involves only some of the layers [1].

Superficial temporal artery aneurysms are a rare diagnosis with approximately 200 cases described in the literature to date. The first patient was reported by Thomas Bartholin in 1740. The first case series was reported by French surgeon DeSanti in 1884 [2]. Subsequent historical review has identified 386 cases dating back to 1644 [3]. Superficial temporal artery aneurysms are primarily traumatic in etiology with upwards of 75%-95% of cases. Causes include physical altercations and sports-related trauma to the head while playing softball, baseball, basketball, hockey, and paintball [4]. These traumatic related pseudo aneurysms typically present sub acutely in the weeks following the event; however, they have been reported to have formed in as early as 24 hours [5]. Spontaneous true superficial temporal artery aneurysms are more rare at approximately 5% of cases and are associated with atherosclerosis or connective tissue disorders [6,7]. Aneurysms can occur anywhere along the course of the superficial temporal artery, however, they are most frequently found in the anterior branch as it transverses the temporal fascial attachment to the superior temporal line. This is attributed to the exposed position of the artery in this area as it courses over the temporal and frontal bones [7].

Symptoms include a pulsatile, growing, temporal mass with tinnitus. Physical examination often reveals a pulsatile mass over the lateral forehead with abolishment of the pulse on proximal compression. In considering the patient with superficial temporal artery aneurysm, the differential diagnosis should include arteriovenous fistula, facial nerve neuroma, abscess, parotid lesion, or other soft tissue mass [8]. Imaging adjuncts include Doppler ultrasonography, computed tomographic angiography, and digital subtraction angiography, which are not required for diagnosis but may reveal synchronous lesions. Lesions can measure up to 4 x 2 cm in size [9].

This report describes a case of a fusiform superficial temporal artery true aneurysm with several lateral feeding branches. These side branches complicated surgical dissection and excision, necessitating additional suture ligation for hemostasis. This combination of features is the first of its kind described in the literature and reinforces the importance of a strong understanding of surgical and anatomic principles in the approaching lesions of this kind. 

## Case presentation

**Figure 1:**
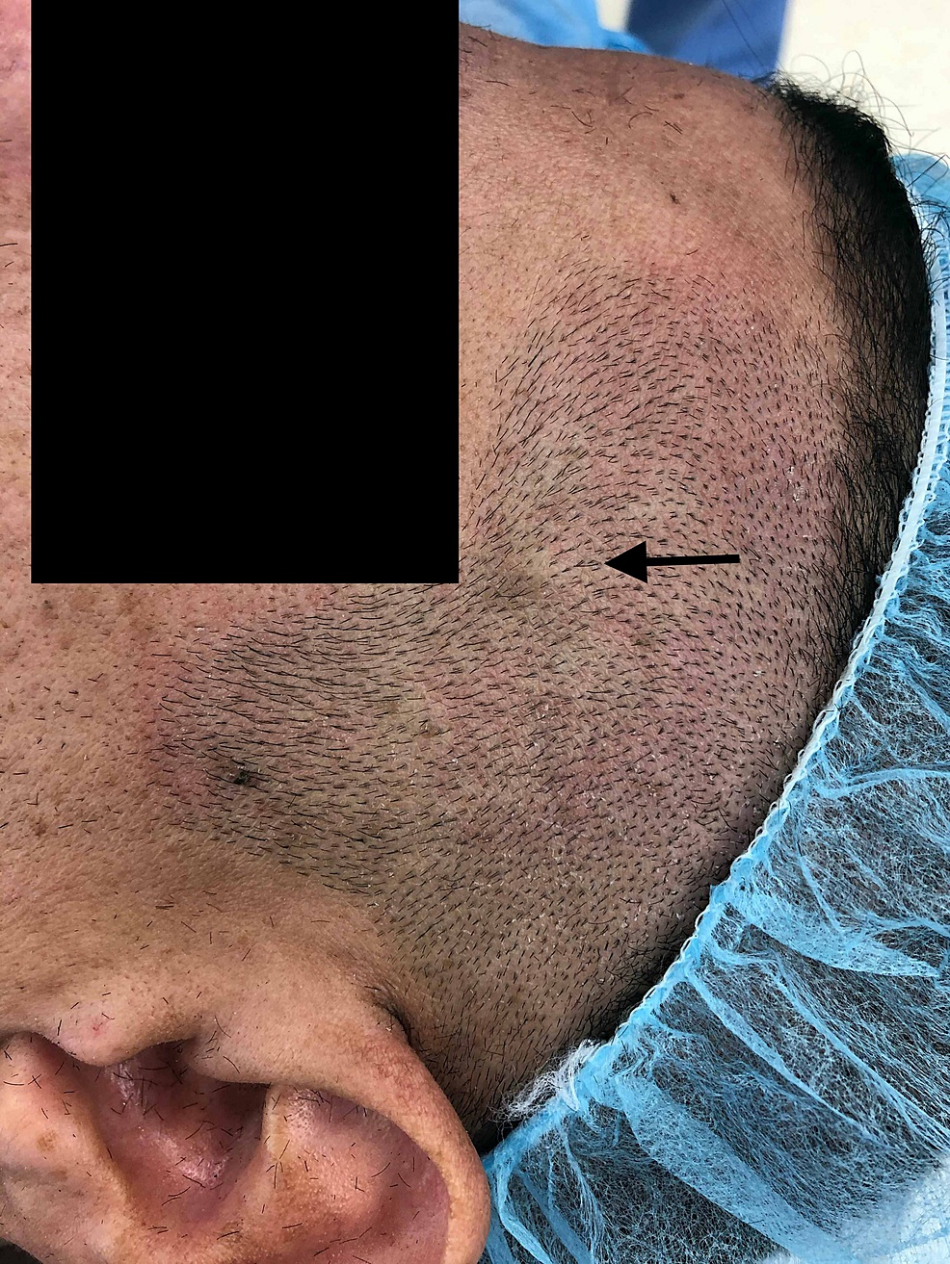
Pulsatile Left Temporal Mass (Arrow) Preoperative Image

A 49-year-old male flight attendant presented with a left forehead mass of six months (Figure 1). He denied tinnitus or audible pulsation in his ear, headache, pain, or other symptoms. The patient reported a past medical history significant for hypertension, pre-diabetes, hyperlipidemia, and gastric bypass 18 months prior resulting in a subsequent 100 lb weight loss. The patient had worked as a flight attendant for the last 25 years on both short and long-haul flights. He denied a history of other aneurysm, hernia, connective tissue disease, or any past trauma to the head. Examination confirmed a pulsatile 1 cm mass overlying the left forehead just within the hairline. Prehospital imaging with computed tomographic angiography confirmed the presence of a fusiform anterior temporal artery aneurysm measuring 6 mm. The patient reported significant anxiety regarding aneurysmal degeneration and the potential for aneurysm rupture while in flight given his profession. The decision was made for surgical management. 

**Figure 2:**
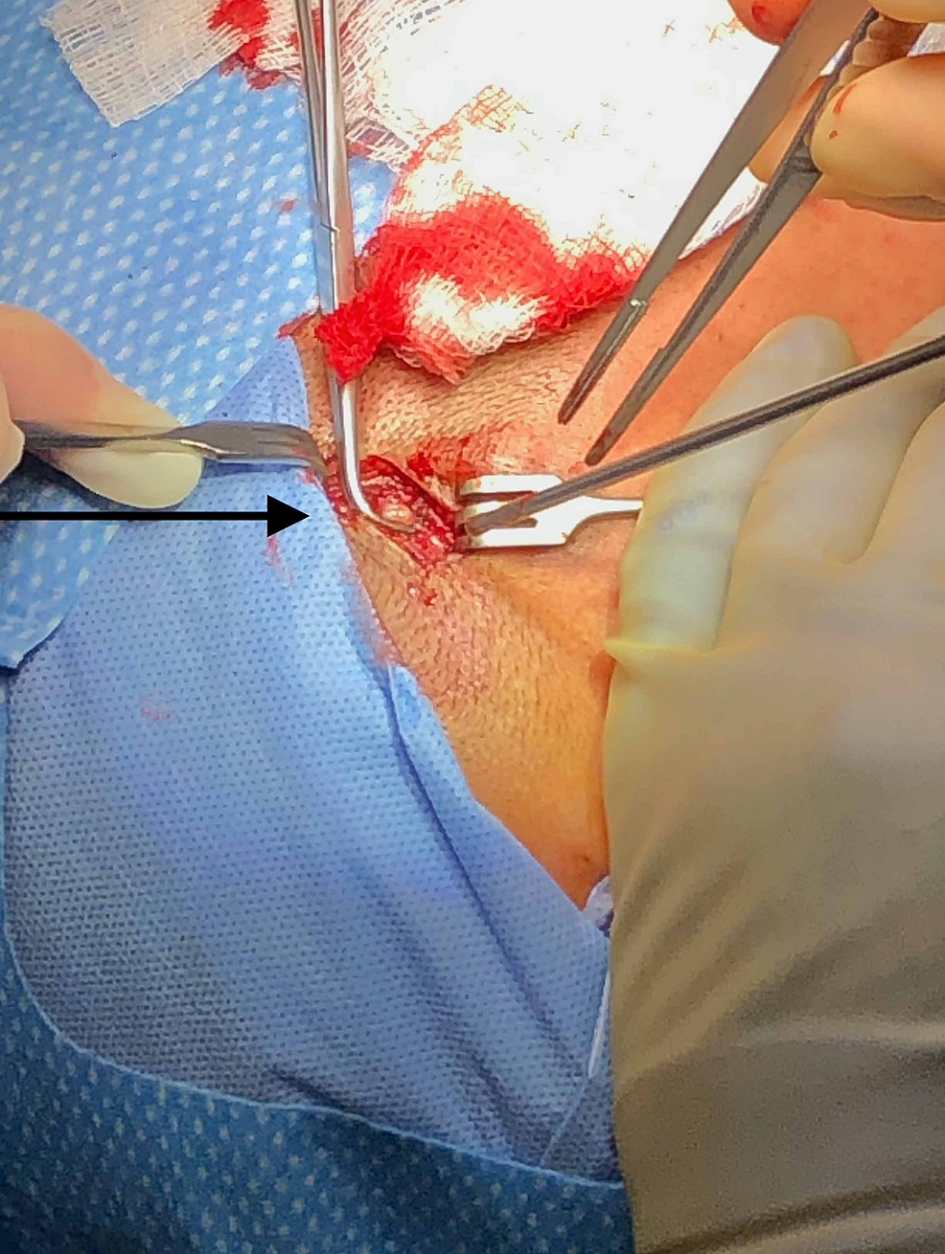
Anterior Superficial Temporal Artery Aneurysm In Situ (Arrow) Proximal and distal control with right angle instrument, enabling further dissection without blood loss.

**Figure 3:**
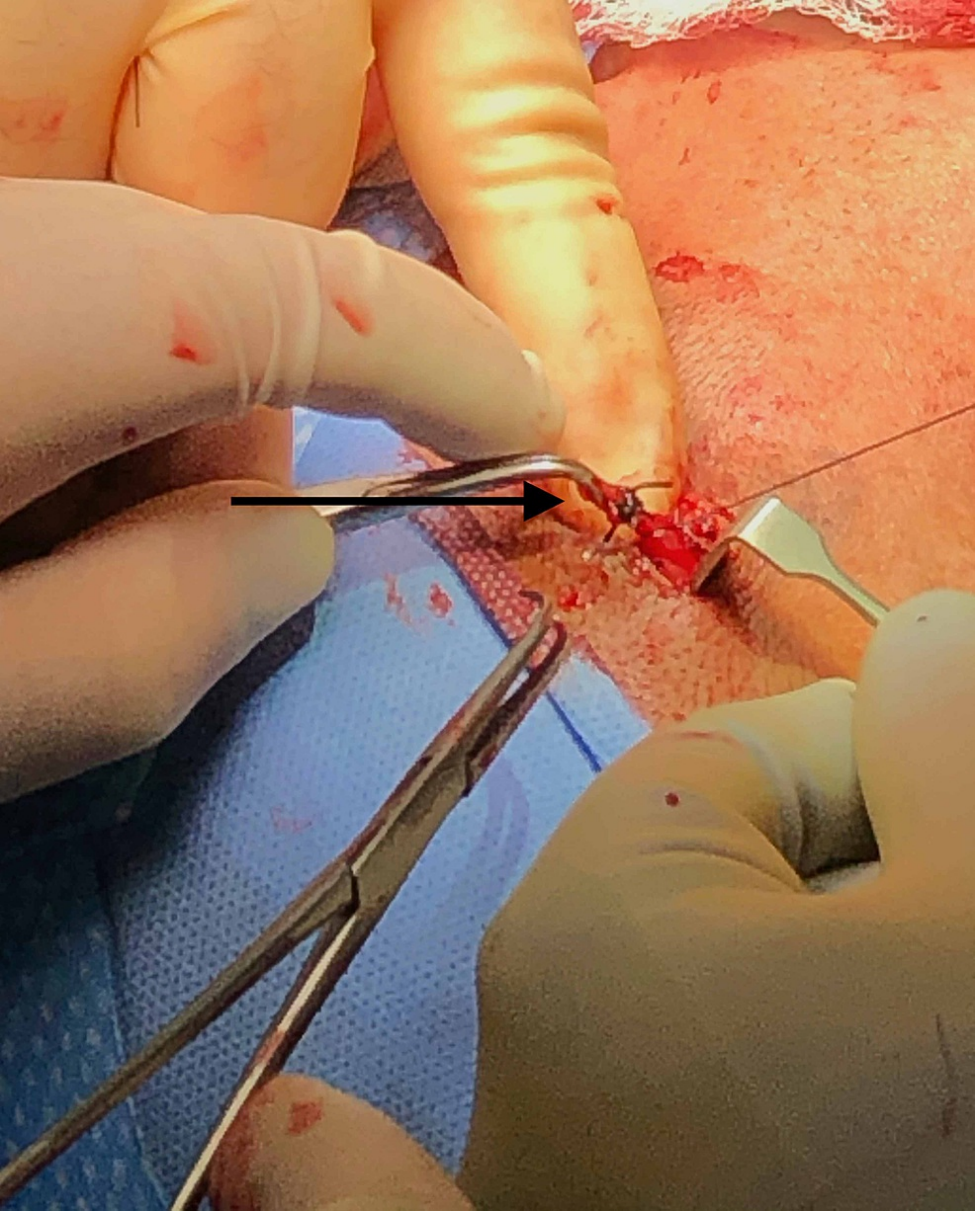
Partially Excised Anterior Superficial Temporal Artery Aneurysm Note fusiform configuration and side branching necessitating a more careful dissection and additional ligation of branch vessels during excision.

The patient was taken to the operating room and placed under general endotracheal anesthesia. A 3 cm oblique incision was made just inside the hairline just proximal to the mass. The dissection was carried down to the temporal fascia, at which point the dissection continued anterosuperiorally until the aneurysm was reached (Figure 2). The aneurysm was bluntly dissected with the proximal and distal ends suture ligated. Several lateral feeding branches were identified during the excision that were suture ligated as well (Figure 3).

**Figure 4:**
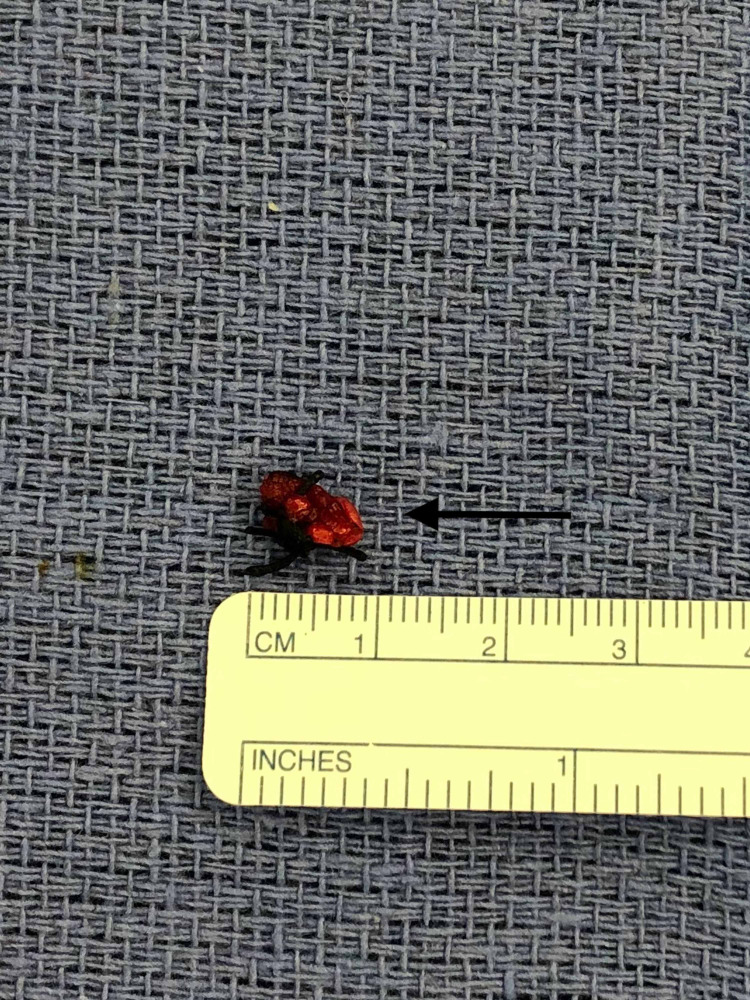
Anterior Superficial Temporal Artery Aneurysm Gross Specimen (Arrow)

The excised aneurysm was found to be 1 cm in diameter and sent for pathologic analysis (Figure 4). The wound was closed using absorbable suture and skin glue. The patient was extubated and taken to the post anesthesia recovery area prior to being discharged home without incident. Pathology of the specimen revealed a medium sized artery and skeletal muscle with aneurysmal dilation of all layers consistent with true aneurysm. Elastin stain revealed an intact lamina elastica, negative for temporal arteritis.

## Discussion

Surgical management of superficial temporal artery aneurysms is recommended as the aneurysm may continue to degenerate over time and burst, presenting with severe hemorrhage [10]. Standard accepted practice consists of open resection of the aneurysmal sac with double suture ligation of the proximal and distal ends. In select cases, the aneurysm may be repaired primarily [11,12]. Endovascular management has also been described with ultrasound-guided direct thrombin injection [13] or embolization and coiling [14]. Endovascular intervention is preferred in the case of facial nerve and parotid gland proximity [8]. Given the superficial location of the artery, surgical management can generally be performed under local anesthesia and moderate sedation. 

Though the majority of temporal artery aneurysms are found to be pseudoaneurysms, which do not involve all layers of the vessel wall, this case was found to be a true aneurysm. The patient denied a social history of smoking and family history of connective tissue disorders, conditions associated with aneurysm formation. He did have a history of hypertension, a risk factor for cerebral aneurysms, and atherosclerosis which has been described in the literature as a potential cause of superficial temporal artery aneurysm [15]. Atherosclerosis is associated with a thinning of the tunica media layer of the vessel wall, leading to wall weakness and aneurysmal degeneration [1]. Finally, as a flight attendant for the past 25 years, the patient was exposed to repetitive cycles of pressurization and depressurization and acceleration and deceleration during flight take-off and landing. These cycles are associated with transient compensatory increases in blood pressure and resultant tension on the arterial wall, which over time may contribute to aneurysmal degeneration [16,17].

An additional unique feature encountered in this case was the lateral branches encountered that continued to feed the superficial temporal artery aneurysm after proximal and distal control had been achieved. This may be due to the more distal location of the anterior superficial temporal artery aneurysm in this case, further from its branch point with the posterior superficial temporal artery. Another factor at play, in this case, is the patient's history of morbid obesity with associated small vessel hypertrophy; changes which persist following massive weight loss [18].

## Conclusions

A 49-year-old man flight attendant with a past medical history of Roux-en-Y bypass and massive weight loss 18 months prior was referred for surgical management of a superficial temporal artery aneurysm. Imaging confirmed the diagnosis. Intraoperatively numerous side branches were identified and controlled. The aneurysm was suture ligated and excised in toto. Pathologic analysis revealed a true aneurysm, only seen in 5% of temporal artery aneurysms. These findings were attributed to the patient's history of morbid obesity and atherosclerosis, with resultant vascular changes that persisted following his massive weight loss, as well as his occupational exposure to repetitive cycles of endovascular stress associated with take-off and landing as a flight attendant. This combination of features is the first of its kind described in the literature and reinforces the importance of a strong understanding of environmental exposures and their compensatory physiologic changes, as well as surgical and anatomic principles to successfully approach and manage lesions of this kind. 
